# Treatment-Resistant Depression in Portugal: Perspective From Psychiatry Experts

**DOI:** 10.3389/fpsyt.2022.824919

**Published:** 2022-03-30

**Authors:** João M. Bessa, Serafim Carvalho, Inês B. Cunha, Milene Fernandes, Ana Matos-Pires, Rui Neves, Albino J. Oliveira-Maia, Susana Santos, Vítor Santos

**Affiliations:** ^1^School of Medicine, Life and Health Sciences Research Institute (ICVS), University of Minho, Braga and Guimarães, Portugal; ^2^ICVS/3B's, PT Government Associate Laboratory, Braga and Guimarães, Portugal; ^3^Clinical Academic Center – Braga, Braga, Portugal; ^4^Department of Psychiatry, Hospital de Magalhães Lemos, Porto, Portugal; ^5^Department of Psychiatry, Hospital Júlio de Matos - Psychiatric Hospital Centre of Lisbon, Lisbon, Portugal; ^6^Real World Evidence and Late Phase, CTI Clinical Trial and Consulting Services, Lisbon, Portugal; ^7^Department of Psychiatry, Unidade Local de Saúde do Baixo Alentejo, Beja, Portugal; ^8^Comprehensive Health Research Center (CHRC), NOVA Medical School - NMS, Universidade Nova de Lisboa, Lisboa, Portugal; ^9^Casa de Saúde da Idanha, Instituto das Irmãs Hospitaleiras do Sagrado Coração de Jesus, Lisbon, Portugal; ^10^Champalimaud Research and Clinical Centre, Champalimaud Foundation, Lisbon, Portugal; ^11^NOVA Medical School - NMS, Universidade Nova de Lisboa, Lisbon, Portugal; ^12^Janssen-Cilag Farmacêutica Lda, Porto Salvo, Portugal; ^13^Department of Psychiatry, Centro Hospitalar e Universitário de Coimbra, Coimbra, Portugal

**Keywords:** treatment-resistant depression, major depressive disorder, patient journey, expert opinion, Portugal

## Abstract

Guidance about treatment-resistant depression (TRD) in Portugal is very limited, even though depression prevalence is among the highest in European countries. A questionnaire was conducted, followed by two advisory boards with seven Portuguese psychiatry experts, to characterize and discuss MDD and TRD epidemiology, diagnosis, patient journey, treatment options, and unmet clinical needs. Consensus was reached on the main issues. In daily practice, TRD can be defined as moderate to severe MDD episodes with insufficient clinical improvement after two antidepressant treatments, taken in adequate doses and duration. TRD diagnosis and treatment are mostly decided by psychiatrists at public hospitals. Treatment type and duration must be adjusted to characteristics of the patient and the depressive episode, including symptoms, number of previous episodes, comorbidities, and previous treatment response and side effects. The most relevant objectives of TRD treatment are reaching response and remission, prevention of suicide, and improvement of quality of life, functionality, and wellbeing. Regarding pharmacotherapy, antidepressant switch occurs more frequently with non-response, while optimization, combination, and augmentation are considered for patients with partial response. Psychotherapy should be considered in parallel to pharmacological treatment. Brain stimulation techniques are underused. Lifelong treatment is required for recurrent or more chronic TRD episodes, but patient adherence is also poorer in these cases. In Portugal, TRD management is limited by lack of access to specialist care and to many treatment options. These aspects highlight that conventional pharmacotherapy does not lead to remission in many patients and that optimization strategies are frequently necessary to achieve satisfactory treatment outcomes.

## Introduction

Depressive disorders are among the most frequent psychiatric disorders, with a prevalence of 4.4% worldwide (1), and around 7–9% in Portugal, which is one of the most affected European countries (2–4). In addition to the high prevalence and associated burden, treating major depressive disorder (MDD) presents several challenges due to its heterogenous manifestations, existence of comorbidities, and the variability and unpredictable nature of response to treatment (5). As a result, ~50–80% of treated patients are reported to have a recurring episode throughout their lives, with only 30–45% of patients reaching complete remission of symptoms after first-line antidepressant treatment (6, 7). The *Sequenced Treatment Alternatives to Relieve Depression* (STAR^*^D) study is the most comprehensive assessment of MDD treatment outcomes, consisting of a randomized controlled trial that ran between July 2001 and September 2006, and providing a demonstration of the latter point (8). The remission rate after two, three, and four sequential trials of antidepressants was 30.6, 13.7, and 13.0%, respectively. Other European studies also included inpatients, resulting in lower rates of remission (9). Hence, although antidepressant drugs have repeatedly been shown to be very effective in several meta-analyses (10), these results demonstrate that they fail to achieve remission in up to one-third of MDD patients (8).

Treatment-resistant depression (TRD) has been defined as a disorder where a moderate to severe MDD episode does not respond to at least two different treatments with antidepressants, at an appropriate dose and treatment duration (9, 11–14), but still with some debate regarding this definition (11, 15). Nevertheless, when compared to treatment-responsive MDD episodes, TRD was associated with higher impact on daily activities, family relationships, and quality of life, in addition to a higher risk of suicide and higher treatment costs (16–18). In fact, some clinical manifestations that are frequently observed in TRD patients (e.g., suicidality, psychotic features, comorbidity anxiety, and among others) complicate patient management and limit response to treatment (9). The low quality of life and reduced productivity of TRD patients were also observed at the baseline of a recent European cohort study, where 46% of TRD patients had failed three or more drugs during the current episode (19). After 6 months of treatment, only 17% of TRD patients achieved remission and 74% showed no response to treatment (12, 19). Information about MDD and TRD management worldwide is still very limited, including in Portugal, where depressive disorders are the third cause of disability (20). This project aimed to provide a first characterization of the clinical practice in Portugal regarding diagnosis, epidemiology, patient journey, and treatment of MDD patients with TRD episodes.

## Methods

An individual questionnaire was sent in May 2020 to a panel of seven psychiatrists (of about 750 psychiatrists in Portugal) (21) with clinical expertise in the treatment of TRD, as well as academic and health decision experience. The seven experts (JB, SC, IC, AMP, RN, AOM, and VS) estimated to have followed, at public and/or private healthcare settings, a total of 2,858 patients with MDD during 2019, corresponding to a median number per expert of 270 (min–max: 150–468) patients with MDD without TRD and 130 (min–max: 20–200) MDD patients with TRD.

The questionnaire included quantitative and qualitative open questions, regarding definitions and diagnosis, epidemiological estimates, patient journey, treatment strategies, and unmet needs. Standardized mean estimates of proportions were calculated by (1) multiplying the estimates of each expert by their respective number of patients followed (i.e., determining expected number of patients with the variable of interest) and (2) dividing the sum of expected patients with the variable of interest by the total number of patients of all experts. After the descriptive analysis of the questionnaire and identification of the key conclusions, two meetings with all experts were conducted online in December 2020, to discuss the main findings and define consensus, whenever possible. This manuscript presents the conclusions of the questionnaire and advisory boards, framed by the most relevant literature.

## Results and Discussion

### A Pragmatic Approach to MDD and TRD Diagnosis and Treatment Definitions

MDD is a highly heterogenous mood disorder (13, 22). In daily practice, MDD diagnosis results from patient observation and identification of signs and symptoms of depression (depressed mood; anhedonia; fatigue; cognitive, psychomotor, and neurovegetative symptoms, among others), assessment of impact on daily life, and exclusion of other disorders or diseases. In fact, the criteria of the *ICD-10 Classification of Mental and Behavioral Disorders* or *Diagnostic* (23) and the *Statistical Manual of Mental Disorders of the American Psychiatric Association* (DSM-5) (24) are important for the classification and validation of the diagnosis (e.g., in the context of clinical studies) but, in daily practice, MDD diagnosis is not strictly bound to verification of these criteria.

Another complexity of MDD management is related to episodes with an inadequate response to treatment or that fail to achieve remission. The lack of a consensus around an operational definition for TRD weakens the generalization of TRD recommendations, despite its burden on patient, caregivers, and services (11, 25, 26). In the European regulatory setting, TRD is defined as an MDD episode for which “treatment with at least two different antidepressant agents (of the same or a different class), prescribed in adequate dosages for adequate duration and with adequate affirmation of treatment adherence, showed lack of clinically meaningful improvement” (27). The experts agreed that TRD definition should refer to the index major depressive episode, detail its severity, and consider other treatment modalities besides antidepressant medication. Hence, from a pragmatic perspective, TRD can be defined as moderate to severe MDD episode with insufficient clinical improvement after two antidepressant treatments taken in adequate doses and duration. Furthermore, the assessment of clinically significant improvement and of episode severity should consider the global assessment of the patient, clinical history, level of disability, and nature of the symptoms that remain after treatment, with particular attention to suicidal ideation, psychotic symptoms, and psychomotor inhibition.

A more global assessment should also be reflected on the assessment of treatment outcomes. Experts agreed with the proposed definitions for remission, i.e., regression of depressive symptoms and return to premorbid functioning, and response, i.e., the occurrence of a substantial clinical improvement that may or may not reach remission (28). However, the definition of these outcomes should extend beyond a defined cutoff in instruments assessing episode severity (overall and in terms of baseline and persistent symptom dimensions). The experts were aware of other proposed concepts, namely, “difficult-to-treat depression”, that integrates a comprehensive and patient-centered assessment of treatment barriers, the illness, and the treatment (29). Importantly, the experts reported that their practice on TRD management already reflects a comprehensive assessment of the patient/episode/treatment triad, although laboratory tests and biomarker determination are not routinely performed in clinical practice across centers (30).

### TRD Patient Profile and Patient Journey

The experts estimated a point prevalence of 9.7% for MDD, which agrees with the data published for the adult Portuguese population (3, 21, 31), with additional estimates that ~32% of patients in Portugal would develop a TRD episode during the course of MDD. This proportion is consistent with the 29% of patients with TRD observed in the previous 12 months as per the experts' estimate (830 TRD cases among 2,858 patients with MDD) and, after adjusting for the number of patients observed by each expert, with the 42% of patients that failed to respond to at least two treatments during the last MDD episode. This TRD estimate of 29–42% is similar to those reported from UK studies (32), from the multicenter *European Group for Study of Depression* trial (33), and from the STAR^*^D trial (6, 34).

Regarding patients with MDD observed during the previous 12 months, the experts estimated that 64% of the patients without TRD episodes were female, with a mean age of 47 years (25% were 65 years or older), and 30% were new diagnoses of MDD. When considering patients with at least one TRD episode during the same period, the experts estimated that 56% were female, aged 52 years old on average (34% were 65 years or older), and 34% were new diagnoses of TRD. Observational studies from other counties have shown similar mean age and proportion of female patients (19, 35, 36). Among factors associated with TRD, the experts acknowledge those reported in the literature (25, 26, 37, 38), and highlighted the characteristics of depression itself (e.g., number of episodes, failure to achieve total remission between episodes, type of depression, persistence of symptoms), having had stressful life events and traumatic experiences (especially during childhood), psychiatric comorbidities (e.g., anxiety and substance abuse), and non-psychiatric comorbidities (e.g., cancer), besides socioeconomic factors (e.g., unemployment), potential genetic determinants (39), and neural biomarkers (40).

The panel estimated that MDD diagnosis is first done on average 11 months (min–max: 6–24 months) after onset of symptoms, suggesting an improvement from the 4 years reported in a 2008 national survey (41). At the index TRD episode, the median time from the onset of symptoms to the recognition of treatment resistance was estimated as 12 months, similar to that observed in UK (42). While it was acknowledged that TRD can be diagnosed during the first episode of MDD, the index episode of TRD was proposed to occur most often after other non-TRD episodes MDD. This estimate of an interval between MDD diagnosis and first episode of TRD is aligned with the 13.7 ± 11.2 months reported by others (19).

**Figure 1:**
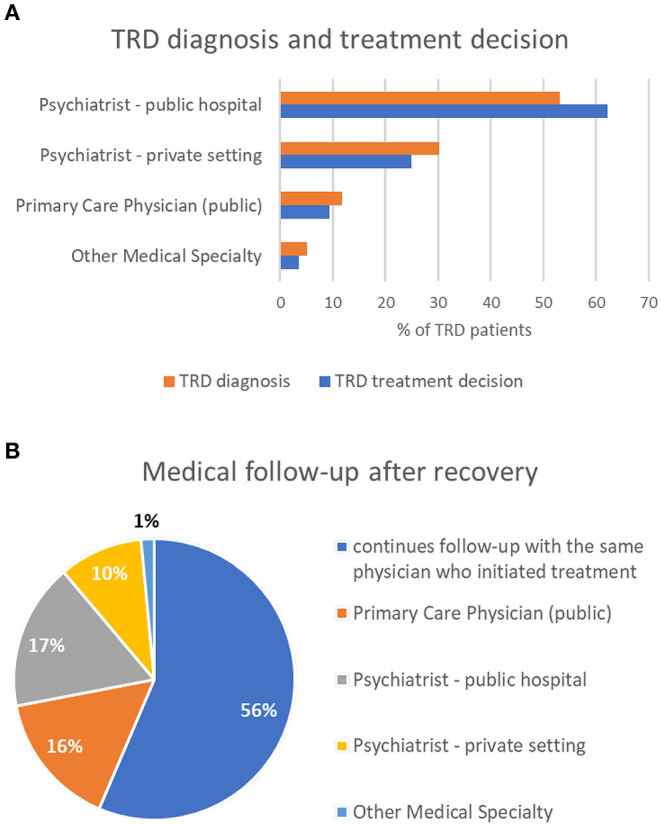
TRD diagnosis and treatment decision **(A)** and follow-up after recovery **(B)**.

The experts also predicted that the majority of TRD diagnoses (83%) and treatment initiation (87%) are made by psychiatrists, namely, at public hospitals (Figure 1), and that most patients continue follow-up with the same physician until remission/recovery (73%) and after recovery (56%). TRD patients are referred to psychiatry outpatient care at public hospitals mainly from primary healthcare units, often before the criteria of resistance to treatment are observed. Furthermore, the experts estimated that patients with TRD referred by other physicians (non-psychiatrists) were frequently under antidepressant medication (81%) but that only 23% were identified with TRD and 32% have never had a prior psychiatry appointment.

### Strategies for MDD and TRD Treatment

The experts acknowledge the relevance of guidelines on MDD treatment such as those from the *National Institute for Health and Care Excellence*, the *Canadian Network for Mood and Anxiety Treatments*, and the *American Psychiatric Association* (13, 43, 44). Other guidelines also referred a hierarchy of treatment strategies and proposed treatment algorithms (45, 46). However, in daily practice, guidelines and therapeutic recommendations must be integrated with clinical experience and adapted to the characteristics of each patient with MDD, especially in TRD episodes. Hence, the choice of an antidepressant for MDD treatment must consider the patient's symptomatic profile, psychiatric and non-psychiatric comorbidities, as well as response to previous treatments and possible side effects. The experts identified the following intrinsic factors as having the greatest impact on the management of MDD, here listed in decreasing order of relevance: suicide risk, severity of the depressive episode, psychotic symptoms or personality disorder, stressful life events, and episode characteristics. Substance abuse and other significant medical conditions, namely, neurological disease and cancer, were also identified as the most challenging comorbidities when treating depressive episodes, and often require a multidisciplinary approach with other medical specialists. These factors have also been described by others, namely, when considering the need for hospitalization (26).

Regarding MDD treatment options in Portugal, antidepressant monotherapy is the most frequent strategy for the first two lines on treatment. As a general guidance, most experts refer that MDD episodes with greater agitation/anxiety component show better results with first-line treatment based on serotonergic drugs, while episodes with melancholic/slowing symptoms may benefit more from treatment with noradrenergic or dual agents. Tricyclic antidepressants were viewed as an option after insufficient response to more recent antidepressants, namely, selective serotonin reuptake inhibitors (SSRIs), serotonin norepinephrine reuptake inhibitors (SNRIs), or dual inhibitors. When considering treatment of TRD episodes, the experts reinforced that there is no defined strategy but rather that the therapeutic decision should depend on the patient and episode characteristics, comorbidities, level of response to previous therapies, side effects, and non-adherence. In their practice, antidepressant switch is the most frequent option after non-response, while optimization, combination, and augmentation (by decreasing order of frequency) are the usual options in cases of partial response.

The perspective of experts on MDD and TRD treatment is consistent with some registry-based studies that identified monotherapy with SSRIs followed by SNRIs as the most frequent options for the first lines of MDD therapy (19, 36, 47). Other guidelines proposed a similar rationale for deciding switching vs. optimization/combination/augmentation strategies, according to the level of previous treatment responses (26, 43–45). However, the available evidence suggests that further research is necessary to define the most appropriate treatment pathways for TRD episodes (48).

Non-pharmacological strategies were also debated. Psychotherapy—among which cognitive-behavioral techniques are the most frequently used—is considered as a parallel axis to pharmacological strategies, being strongly recommended to be carried out by psychiatrists or psychologists with appropriate training. The use of brain stimulation techniques should be considered as a treatment option for TRD, depending on the severity of the depressive episode, previous and current non-response or side effects of the medication, as well as patient choice. It was also noted that, in the pandemic context in Portugal, accessibility to brain stimulation worsened considerably. Other studies indicated that neurostimulation techniques are underused, including in TRD episodes (19, 36, 49).

### Treatment Objectives and Response/Remission Estimates From the Clinical Practice

For the experts, the main treatment objectives are reaching response and remission, prevention of suicide, and improvement of quality of life, functionality, self-perceived wellbeing, and family relationships. Individual definition of these goals is based on unstructured patient observation and assessment, with rating scales most often used for monitoring treatment with brain stimulation techniques and in more severe cases.

**Figure 2:**
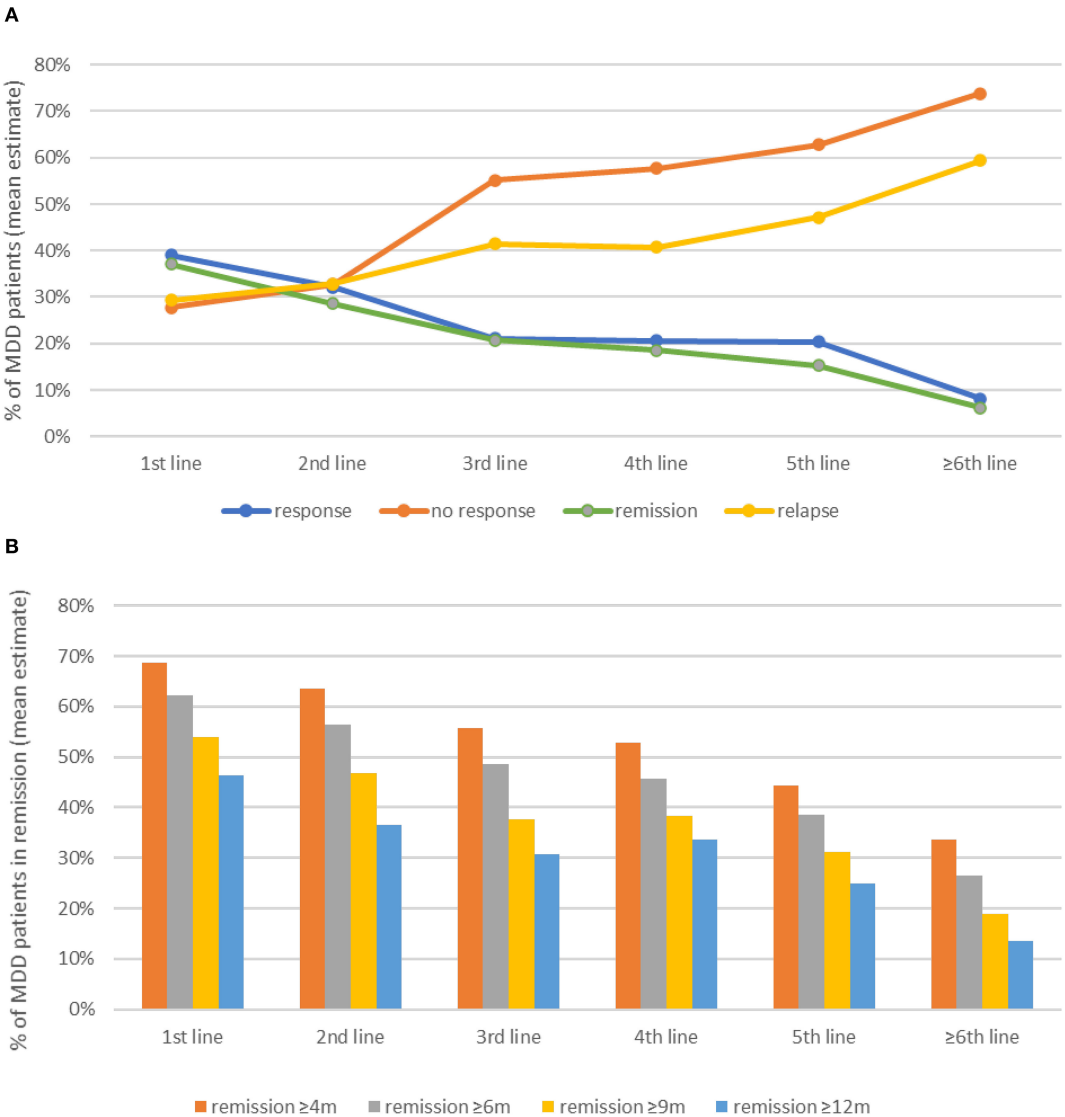
Rates of response and remission **(A)** and duration of remission after successful treatment **(B)**.

Overall, rates of response, remission, and maintenance of remission decrease with the progression of therapeutic lines, especially in the context of pharmacological treatment (Figure 2). When the remission is not achieved, potential pseudo-resistance—by insufficient plasma levels, patient non-compliance, or relevant psychiatric and/or somatic comorbidities—has to be excluded before treatment optimization (45, 46). In line with other recommendations (26), maintenance treatment is extended in recurrent episodes (compared to first episodes without risk of recurrence) and for patients with TRD episodes: up to 36 months for patients with response to a 3rd or 4th line of treatment and lifelong for patients on a 5th or later line of treatment. However, the experts also estimate that patients leave treatment more frequently with the progression of therapeutic lines and mostly in the maintenance phase, with loss to follow-up of 32–40% vs. 12–28% of patients during acute treatment. Psychoeducation, the involvement of caregivers, and a strong patient–physician relationship are seen as crucial to promote adherence to treatment.

## Conclusion

The high number of relapses, poor adherence to treatment, difficult access to psychotherapy, and other non-pharmacological treatment strategies and insufficient efficacy of available medication for treatment of TRD were identified by the experts as the main barriers to treatment success. In fact, even though antidepressants have proven their efficacy on MDD treatment, TRD episodes, frequently recurrent, are still a challenge for clinical practice (50). These aspects highlight that conventional psychopharmacotherapy does not lead to remission in every patient and that optimization strategies are frequently necessary to achieve satisfactory treatment outcome, whereby recent international recommendations may further contribute to successful treatment (45). TRD is a high burden for patients, caregivers, and healthcare services, and there is a need for improvement of access to treatment options that provide sustained responses, as well as development of novel therapeutic options for the future (17, 51).

Some limitations are acknowledged, as this was an advisory board, based on expert consensus and opinion, rather than strong data from an empirical study. Nevertheless, care was taken to reduce potential bias from dominant opinions among experts, namely, through the use of the initial individual questionnaire, assuring that answers from all experts were considered. It is also possible that the perspective of the experts involved here may lack the insight of other clinicians involved in MDD and TRD management, namely, primary care physicians. Nevertheless, there is some consensus that, in Portugal, TRD diagnosis and treatment are usually performed by psychiatrists. Hence, based on the clinical experience of the experts involved here, as well as data available in the literature, this manuscript provides an insight into the Portuguese context of MDD management, while also providing estimates of clinical characteristics and treatment results in the context of TRD.

Overall, the expert consensus was consistent with observational studies and recommendations that have started to unveil the barriers to successful treatment of TRD episodes (12, 14), which were augmented by the COVID-19 pandemic (52, 53). Mental health services and MDD management in Portugal require an urgent investment, namely, by providing patients with facilitated access to available treatment options, including psychotherapy, neurostimulation, and novel pharmacological strategies.

## Data Availability Statement

The original contributions presented in the study are included in the article/supplementary material, further inquiries can be directed to the corresponding author.

## Author Contributions

JB, SC, IC, AM-P, RN, AO-M, and VS: expert participation and critical revision of the manuscript. SS: questionnaire development and revision of the manuscript. MF: questionnaire development, data analysis, and drafting the manuscript. All authors revised the manuscript draft and approved the submitted version.

## Funding

Support for third-party advisory board logistics and writing assistance, provided by CTI Clinical Trial and Consulting Services, was funded by Janssen-Cilag Farmacêutica Lda in accordance with Good Publication Practice (GPP3) guidelines.

## Conflict of Interest

SS is an employee of Janssen-Cilag Farmacêutica Lda. MF is an employee of CTI Clinical Trial and Consulting Services. The remaining authors received advisory board fees from Janssen-Cilag Farmacêutica Lda. AO-M was the national coordinator for Portugal of a non-interventional study EDMS-ERI-143085581, 4.0 to characterize a Treatment-Resistant Depression Cohort in Europe, sponsored by Janssen-Cilag, Ltd. 2019–2020; is the recipient of a grant from Schuhfried GmBH for norming and validation of cognitive tests; and is the national coordinator for Portugal of trials of psilocybin therapy for treatment-resistant depression, sponsored by Compass Pathways, Ltd. EudraCT numbers 2017-003288-36 and 2020-001348-25, and of esketamine for treatment-resistant depression, sponsored by Janssen-Cilag, Ltd. EudraCT Number: 2019-002992-33. Janssen had no influence on the interpretation of results. This manuscript presents the opinion of the psychiatry experts only.

## Publisher's Note

All claims expressed in this article are solely those of the authors and do not necessarily represent those of their affiliated organizations, or those of the publisher, the editors and the reviewers. Any product that may be evaluated in this article, or claim that may be made by its manufacturer, is not guaranteed or endorsed by the publisher.
